# Primary Sclerosing Cholangitis Associated with Elevated Immunoglobulin-G4: A Preliminary Study

**DOI:** 10.5402/2012/325743

**Published:** 2012-09-04

**Authors:** Baran Parhizkar, Amir Houshang Mohammad Alizadeh, Hamid Asadzadeh Aghdaee, Habib Malekpour, Amir Hossein Entezari

**Affiliations:** Research Center for Gastroenterology and Liver Disease, Taleghani Educational Hospital, Shahid Beheshti University of Medical Sciences, Tehran 19857, Iran

## Abstract

*Background*. Immunoglobulin IgG4-associated cholangitis (IAC) disease is a systemic disease histologically characterized by extensive T lymphocytes and IgG4 positive plasma cell infiltration in various organs. Prevalence of IAC in PSC patients was reported to be between 7% and 11.6% in a few previous studies. This study was carried out to evaluate frequency of serum IgG4 level in PSC patient referred to the gastroenterology ward of Taleghani educational hospital in Tehran, Iran. *Material and Methods*. This study was a prospective analytical cross-sectional study. Clinical presentation, laboratory values, imaging changes, inflammatory bowel disease (IBD), esophageal varices, ascites, and child score in newly PSC patients with elevated IgG4 were determined and compared with PSC patients with normal levels of IgG4. Data was analyzed by using SPSS software. The frequency and standard deviations were calculated. Differences among groups were evaluated by using the chi-square, fisher exact, and Mann-Whitney *U* tests. *Results*. 34 patients with PSC were examined in the study period, of which 9 cases (26.5%) had high IgG4 levels. Most of the patients were male, 23 cases (67.6%) and nonsmoker, 26 cases (76.5%). Patient average age was 47 years old (range 21–67 years). There was not any significant relationship among patients with IAC and PSC patients in terms of variables such as age, smoking, presence of IBD, ascites, esophageal varices, child score, and imaging findings (*P* > 0.05). *Conclusion*. IAC should be suspected in cases of unexplained biliary strictures with increased serum IgG4. Testing PSC patients for IgG4 and treating those who have high levels with corticosteroids in clinical trials should be considered in future studies.

## 1. Introduction

Primary sclerosing cholangitis (PSC) is a chronic cholestatic disease of intrahepatic and extrahepatic biliary ducts, characterized by chronic periductal inflammation and sclerosing of the ducts, which results in segmental stenosis of bile ducts, cholestasis, and fibrosis [1].

During two last decades, patients with steroid responsive PSC have been presented in some case reports and a few studies [2].

In 1999, Erkelens et al. [3] described 4 cases of PSC with good response to corticosteroid therapy; therefor, posing idea of presumable autoimmune basis for this type of sclerosing cholangitis and it is named later Immunoglobulin G4- (IgG4-) Associated Cholangitis (IAC) [2].

IAC disease is a systemic disease histologically characterized by extensive T lymphocytes and IgG4 positive plasma cell infiltration of various organs [4]. Most frequent clinical signs and symptoms at presentation included jaundice (77%), weight loss (51%), and abdominal pain (26%) [1]. Despite of increasing recognition of IAC as an independent disease entity, its pathogenesis still remains unknown [4], and the sensitivity and specificity of IgG4 levels for the diagnosis of IAC are not known, at present time [2]. 

Prevalence of IAC in PSC patients reported between 7% and 11.6% in a few previous studies [2, 5, 6].

This study was carried out to evaluate frequency of serum IgG4 level in PSC patient referred to referral gastroenterology ward of Taleghani educational hospital in Tehran/Iran for the first time. 

## 2. Material and Methods

This study was a cross-sectional prospective, descriptive and analytical study on 34 patients with PSC (23 were male and 11 were female with median age 43.2 years (range 21–67 years)). Newly PSC patients which were diagnosed according to the (1) chronic cholestasis of unknown etiology for at least 6 months, (2) biliary strictures typical for PSC was seen in cholangiography (MRCP or ERCP) by observation of beading appearance, and (3) appropriate exclusion of other hepatic or biliary disease using standard clinical, imaging, and laboratory criteria were included in the study by census method in Tehran Taleghani educational hospital during January, 2011 to January, 2012.

Data on clinical presentation, laboratory values, extent of the cholangiographic changes, inflammatory bowel disease (IBD), esophageal varices, ascites, and child score was collected in questioners. The IgG4 levels, CA 19-9 antigen, AMA, ASMA, and ALKM antibodies were measured and recorded in questioner too. 

Patients with elevated IgG4 were determined and their laboratory and other variables were compared with PSC patients with normal levels of IgG4. 

Data was analyzed by using SPSS version 16 software. The frequency and standard deviations were calculated. Differences among groups were evaluated by chi-square or fisher exact tests for qualitative variables, and nonparametric quantitative variables (such as median IgG4 levels) were compared by Mann-Whitney *U* test.

## 3. Results

 Thirty-four patients with PSC were examined in the study period; that 9 cases (26.5%) had high IgG4 level (>157 mg/dL, normal range 10–157 mg/dL). The mean of IgG4 levels in IAC patients was 218.00 ± 46.33 (159–292 mg/dL). Out of 34 cases, 23 of them were male (67.6%) and 26 cases were nonsmoker (76.5%). 

There was not any significant relationship among patients with IAC and PSC patients in terms of variables such as age, smoking, presence of IBD, ascites, esophageal varices, child score, and laboratory and imaging findings (*P* > 0.05) (Table 1).

33.3% of IAC patients and 44% of PSC patients with normal IgG4 levels had IBD (*P* > 0.05).

Intrahepatic involvement and extrahepatic involvement were seen in 55.6% and 11.1% of IAC patients, and in 33.3% of them both intra- and extrahepatic involvement were seen (*P* > 0.05).

High CA19-9 was seen in 77.8% of IAC patients. Serum value of CA19-9 among patients with PSC was between 11 and 347, and 7 patients have high level of CA19-9 (above 347 mg/dL).

Laboratory findings in IAC patients including liver enzyme and serum IgG4 levels, CA 19-9 antigen, AMA, ASMA, and ALKM antibodies are listed in Table 2.

## 4. Discussion

Elevated serum IgG4 levels (IAC) were seen in 26.5% of PSC patients in our study. So far, few studies have been published which evaluated the increase in IgG4 in patients with PSC [2, 7], the highest previous reported prevalence was 11.6% which has been mentioned in the Björnsson et al. study [2]. It seems that our achieved frequency is the highest figure reported in PSC patients with increased IgG4 levels. Meanwhile, a few previous studies evaluated an increase in IgG4 in patients with PSC [2, 7] and other studies have been conducted on patients with AIP [1, 6]. 

Our results suggest that IgG4 measurement should be considered in all patients with PSC and should be a part of the diagnostic criteria in these patients. 

The first case report of IgG4-related systemic disease (ISD-) like disorders date backs to the sixties [8]. 

Two autoimmune and allergic mechanisms are proposed in the pathogenesis of IAC [4], but whether induced responses by T-helper 1 cells (as an autoimmune disease) [9] or dominant semiallergic response of T-helper 2 cells can induce inflammatory reaction in IAC patients is unclear yet [10–12]. 

Increased IgG4 titers can be seen in both types of allergic diseases such as asthma and atopic dermatitis [11, 13] and autoimmune diseases such as pemphigus vulgaris, myastenia gravis, vasculitis, and systemic lupus erythematous [14]. The role of IgG4 in pathogenesis of IAC is not clearly clarified yet.

Now HISORt criteria, which is provided by the Mayo Clinic, has been recognized as the most useful diagnostic criteria of IgG4-SC [1]. 

It is well known that IAC is a manifestation of a systemic fibroinflammatory process involving the bile duct, pancreas, salivary and lacrimal glands, retroperitoneum, and lymph nodes. The association of IAC with autoimmune pancreatitis (AIP) is of particular clinical relevance. Studies from the Mayo Clinic revealed that 92.5% of patients with IAC suffer from AIP whereas IAC may occur in 20%–90% of cases of AIP. In fact, unexplained pancreatic disease and other organ involvement are important clues in the diagnosis of IAC [1].

In Mendes et al. study [6], patients with increased IgG4 levels have higher levels of bilirubin, alkaline phosphatase, and Mayo risk score and less time for liver transplantation. Also in the Björnsson et al. study [2] liver cirrhosis was seen in 50% of patients. How ever, in our study there was not any significant relationship among bilirubin, alkaline phosphatase, AST, ALT levels, and child score in patients with elevated IgG4 levels (IAC) compared to PSC patients with normal IgG4 levels. 

The number of reviewed PSC patients in the Mendes et al. [6] and Björnsson [2] studies was 127 and 285 cases respectively, whereas in our study this figure was 34 cases; it seems, despite of the same age range of our patients and the two previous studies, especially Björnsson et al. study [2], restrictions on the sample size in our study could partly be effective in the absence of statistical difference in factors listed previously.

In some previous studies that have investigated the relationship between laboratory factors and increased serum IgG4 levels [1, 15], patients had a mean age of 60 years. 

On the other hand as we did not include the control in this study and according to our results, we cannot certainly comment on severity of illness in IAC patients compared to those with normal serum IgG4 levels.

In our study there was not any association among clinical statuses, such as the presence of ascites and esophageal varices, with presence of IAC which result is coordinated with the results of previous studies [13, 16]. These findings indicate that there was not any specific clinical sign suggestive for IAC in patients with PSC.

In this study, IBD was seen in 41.2% of all PSC patients and 33.3% of IAC patients. The only previous study that has evaluated IBD in PSC patients with elevated IgG4 was Björnsson et al. study [2], which indicated IBD in 75% of their cases and had higher figure in comparison with our study.

IAC is characterized by bile duct wall thickening and biliary strictures, which are, in contrast to primary sclerosing cholangitis, predominantly located in the lower bile ducts. In a review of 53 patients with IAC, Ghazale et al. [1] reported that IAC is associated with radiologic findings of AIP in 92% and elevated serum IgG4 in 74%. Therefore, more information on the cholangiographic findings might be useful along with a comment on the higher incidence of intrahepatic compared with extrahepatic biliary tract involvement among IAC patients in this study. 

According to the results of this study in comparison with results of other studies [2, 4], it seems that both intra- and extrahepatic involvement should be considered in patients with IAC. 

The important point that should be considered is that cholangiographic changes in IAC, pancreatic cancer, PSC, and cholangiocarcinoma are more similar and if diagnosis of IAC will be neglected, it can lead to invasive intervention for patients. Therefore, based on cholangiographic findings we suggest that evaluation of serum IgG4 is useful in diagnosis of IAC cases and changing therapeutic process in these patients. 

In this regards, Nakazawa and colleges [15] in their study which was published in 2012 evaluated diagnostic criteria of IgG4-related cholangitis based on cholangiographic findings and noticed different diagnostic factors for any of PSC, pancreatic cancer, and cholangiocarcinoma. 

It seems that future studies in this field can be considered as the next research step.

Limited sample size and impossibility for follow-up response to corticosteroid treatment in our patients due to restrictions on access to patients were the major limitation of our study.

Based on the findings of this study, measured serum IgG4 level in all patients with PSC should be considered especially at diagnose time. 

Future studies in this field especially studies with control groups and also multicenter clinical trials in the field of corticosteroid therapy and other useful therapies are suggested.

## Figures and Tables

**Table 1:** Compare demographic characteristics of IAC and PSC with normal IgG4 patients.

	PSC (normal IgG4)		IAC (elevated IgG4)	
	Frequency (*n*)	Percentage (%)	Frequency (*n*)	Percentage (%)
Gender				
Male	16	69.6	7	30.4
Female	9	81.8	2	18.2
Smoking				
Yes	4	50	4	50
No	21	80.8	5	19.2
IBD				
Yes	11	78.6	3	21.4
No	14	70	6	30
Ascites				
Yes	3	100	0	0
No	22	71	9	29
Varices				
Yes	4	80	1	20
No	21	72.4	8	27.6
Child score				
Child A	16	69.6	7	30.4
Child B	6	75	2	25
Child C	3	100	0	0
MRCP				
Intrahepatic	12	70.6	5	29.4
Extrahepatic	4	80	1	20
Both	9	75	3	25

*P* > 0.05 in all variables.

**Table 2:** Demographic clinical, and laboratory features in the IAC patients. Laboratory parameters are demonstrated as median and IQR.

	Patients with PSC with IgG4 elevation (IAC) (*n* = 9)
Age, years	45 (21–60)
Gender (male)	7 (77.8%)
Smoking	4 (44.4%)
IBD Present	3 (33.3%)
High CA 19-9	7 (77.8%)
AMA (±)	0
ASMA (±)	1 (11.1%)
ALKM (±)	0
Intrahepatic involvement (MRCP)	5 (55.6%)
Extrahepatic involvement (MRCP)	1 (11.1%)
IgG4 (10–157 mg/dL)	218.00 ± 46.33 (159–292 mg/dL)
Hb	12.58 ± 1.87 (8–14.4)
AST (<40 U/L)	75.44 ± 51.85 (27–160)
ALT (<40 U/L)	69.88 ± 51.08 (19–160)
Alk Ph (<200 U/L)	664.11 ± 328.29 (327–1436)
Bil total (<2 mg/dL)	2.82 ± 2.53 (0.4–6.90)
Bil direct (<2 mg/dL)	1.84 ± 2.21 (0.1–5.8)
